# Seasonal Variation and Frequency Distribution of Ectoparasites in Crossbreed Cattle in Southeastern Brazil

**DOI:** 10.1155/2014/759854

**Published:** 2014-10-08

**Authors:** Maria do Socorro Ferraz da Costa, Marcos Pezzi Guimarães, Walter dos Santos Lima, Ana Julia Ferraz da Costa, Elias Jorge Facury Filho, Ricardo Nascimento Araujo

**Affiliations:** ^1^Departamento de Parasitologia, Instituto de Ciências Biológicas, Universidade Federal de Minas Gerais, Bloco i4, Sala 177, Avenida Antonio Carlos 6627, Pampulha, 31270-901 Belo Horizonte, MG, Brazil; ^2^Departamento de Clínica de Ruminantes, Escola de Veterinária, Universidade Federal de Minas Gerais, Avenida Antonio Carlos 6627, Pampulha, 31270-901 Belo Horizonte, MG, Brazil; ^3^INCT-Entomologia Molecular, 21941-590 Rio de Janeiro, RJ, Brazil

## Abstract

The aims of this study were to evaluate the seasonal variation and frequency distribution of *Rhipicephalus (Boophilus) microplus*, *Haematobia irritans*, and *Dermatobia hominis* on crossbred heifers under field conditions in the northeast of Minas Gerais state, southeastern Brazil. From November 2007 to September 2009 (23 months), 40 heifers aged 16.6 ± 2.4 months were divided into groups A (1/4 Holstein × 3/4 Gir) and B (1/2 Holstein × 1/2 Gir) and had the monthly infestation estimated along with the climatic conditions. The mean maximum and minimum temperatures were 28.5 and 19°C, respectively. The ectoparasites were present on animals in all months of the year. The levels of ticks on the animals were low (3.0 ± 0.2 ticks/animal), with the highest density in midwinter. The temperature was the climatic factor that most influenced the tick levels. The population of *H. irritans* (13.9 ± 0.3 flies/animal) and *D. hominis *(1.5 ± 0.2 larvae/animal) on heifers was more influenced by rainfall and exhibited two population peaks during the year. 1/2 Holstein heifers harbored significantly more *H. irritans* and *D. hominis *than 1/4 Holstein heifers. The results are discussed considering the most appropriate periods to apply ectoparasiticides and the genetic make-up of the animals.

## 1. Introduction

The ectoparasites of cattle in Brazil are a significant hindrance to national livestock. Among the primary ectoparasites are the tick* Rhipicephalus (Boophilus) microplus* and the flies* Haematobia irritans* and* Dermatobia hominis*. Together, the losses caused by these parasites are estimated at more than US$ 2.5 billion per year [1, 2].

Cattle kept under field conditions tend to be parasitized simultaneously by different species of ectoparasites. Proposals to combat more than one parasite at once are more economical and more operationally feasible and may have greater acceptance by farmers [3]. Most control programs are based primarily on the application of ectoparasiticides. The application of these compounds in a strategic manner reduces the amount of ectoparasiticides used and prevents population peaks, keeping the parasite burdens below the levels that cause economic losses. However, for satisfactory results, the number and timing of applications must take into account the biological and ecological characteristics of each ectoparasite in the region in which the program will be implemented. Another very attractive strategy is the insertion of genes from parasite-resistant breeds to use the animals' immune systems to help fight off the parasites [4]. Several studies have shown that* Bos indicus* is more resistant to ectoparasites than* Bos taurus* and the introduction of* B. indicus* genes into the herd promotes an increase in the resistance of animals against ectoparasites without compromising production [5, 6].

Given the large area covered by Brazil and the variety of cattle breeds raised, it is important that studies performed consider the peculiarities of each physiographic region and the genetic patterns of the animals. Minas Gerais (MG) is one of the major milk-producing states in Brazil with most of the cattle herds among Holstein and Gir breeds and their crosses. In the present study, two groups of heifers with different genetic make-up were used to evaluate three different aspects under field conditions: (a) population levels and seasonal variation of ectoparasites; (b) the frequency distribution of the ectoparasites in the animals of the herd; and (c) the difference of infestation with ectoparasites between the groups with 1/2 and 1/4* B. taurus* genetic make-up. The results may be useful in developing control strategies for use in the studied region or other regions with similar characteristics.

## 2. Materials and Methods

The study was developed on a commercial dairy farm in the municipality of Teófilo Otoni, MG (17°51′15′′ latitude, 41°30′23′′ longitude and 334 meters above sea level), Southeastern Brazil.

Forty heifers aged 16.6 ± 2.4 months were divided into two groups: group A: 32 heifers, 1/4 Holstein ×  3/4 Gir and group B: 8 heifers, 1/2 Holstein ×  1/2 Gir. During the experimental period—November 2007 to September 2009—the animals were kept together in an area of 40 ha consisting of grass* Brachiaria brizantha*. Water and mineral supplementation was offered* ad libitum* and in some months of the dry season animals were supplemented with sugar cane. To avoid animal suffering, ectoparasiticide emergency treatments where applied on each heifer with number of ticks counted on one side of the body ≥40 or number of active nodules caused by* D. hominis* larvae in the whole body ≥20 or number of horn flies counted in the whole body of animals ≥200. The emergency treatments were performed with 0.2% trichlorphon (Neguvon, Bayer) and were carried out always after the parasite counts. The drug was diluted in water and applied topically using a paint brush only where parasites were observed.

The animals were gathered monthly in a corral between 7:00 and 10:00 a.m. and heifers were restrained individually in a chute, held by approximately one minute to let the flies sit and, after then, two trained observers (one in each side of the animal) counted the number of larvae of* D. hominis* and adults of* H. irritans* across the whole body surface of each animal. The number of* R. microplus* greater than 4.5 mm in diameter was counted on the right surface of each heifer and this number was multiplied by two to determine a total of ticks on the host.

Meteorological data including the mean maximum temperature, the mean minimum temperature, and the rainfall level were collected from a weather station located 10 km from the place of the experiment.

Ectoparasite numbers were analyzed using the Kolmogorov-Smirnov test to evaluate normality. Spearman's correlation coefficient was used to assess potential correlations among variables. The two-tail Mann-Whitney *U* test was performed to assess differences between groups A and B. The analyses were carried out using GraphPad Prism 5 (GraphPad, Inc.) software. The level of significance adopted was *P* < 0.05. All data are presented as the mean ± standard error (SE) or median (quartile 1–quartile 3).

## 3. Results

**Figure 1:**
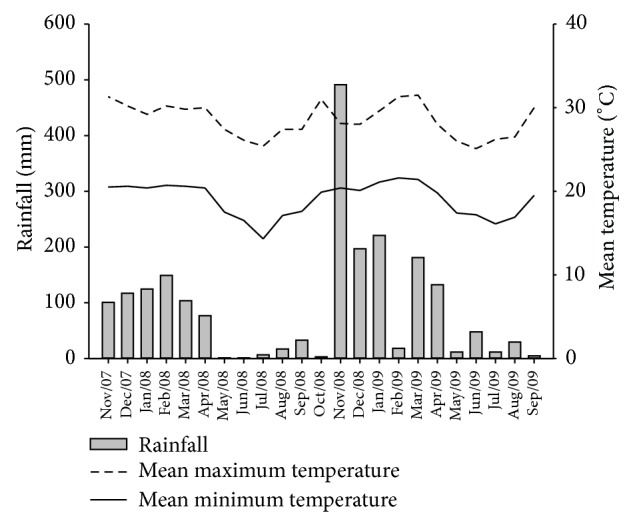
Mean monthly maximum and minimum temperatures and rainfall during the experimental period.

The mean maximum and minimum temperatures during the experimental period were 28.5 and 19°C, respectively. The temperatures were considered high, with the mean maximum temperature above 25°C in all months and exceeding 29°C in 11 of the 23 months analyzed. The mean minimum temperature was below 15°C only in July, 2008. The region was characterized by a rainy season (September to April), with monthly rainfall of up to 491 mm, and a dry season (May to October), with rainfall ranging from 1.2 to 47.8 mm per month (Figure 1).

**Figure 2:**
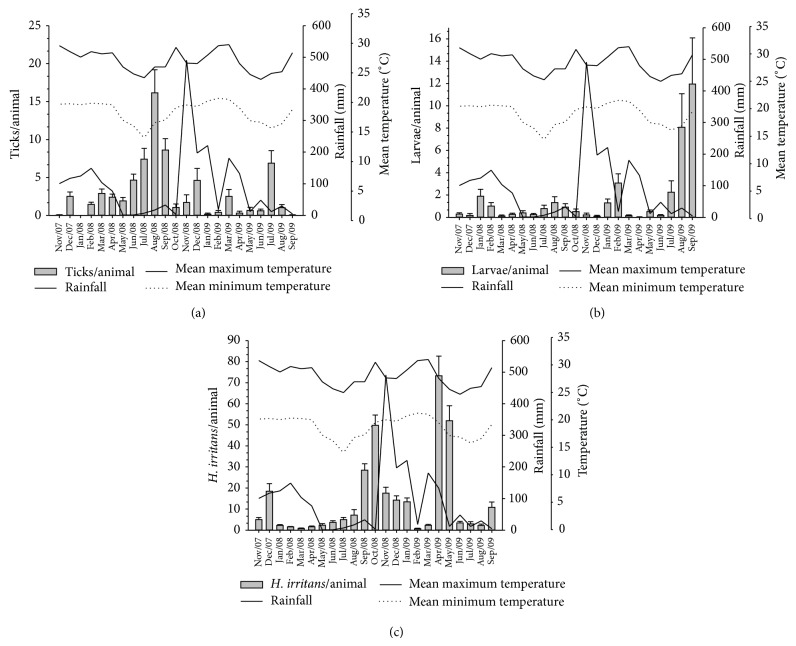
Mean total number (mean ± SE) and seasonal variation of* Rhipicephalus microplus* (a) and larvae of* Dermatobia hominis* (b) and* Haematobia irritans* (c) on heifers from Holstein × Gir crossbreeds from November 2007 to September 2009 in the northeast of Minas Gerais State, Brazil.

The mean number of ticks of the flock throughout the period was 3.0 ± 0.2 per animal, and the monthly means ranged from zero to 16.2 (Figure 2(a)). The highest mean number of ticks per animal occurred in August and September 2008 (16.2 ± 3.0 and 8.6 ± 1.5 ticks/animal, resp.), months when the mean temperatures were between 14 and 28°C. The counts of* R. microplus* exhibited a significant negative correlation (*P* < 0.05) with the mean minimum temperature (*r*_*s*_ = −0.3734; *P* < 0.05) (Table 1).

The mean number of larvae of* D. hominis* observed on animals during the experimental period was 1.5 ± 0.2 (Figure 2(b)). Most monthly botfly infestations were below 2 larvae per animal, except in four months (February, July, August, and September 2009). Two peaks of infestation were observed during the year, the first in January-February (midsummer) and the second in August-September (late winter to early spring). The number of larvae per animal exhibited a significant negative correlation (*r*_*s*_ = −0.3824; *P* < 0.05) with precipitation (Table 1). Other weather parameters showed no significant correlation (*P* > 0.05) with botfly larvae numbers.

*H. irritans* showed an overall mean of 13.9 ± 0.3 flies per animal and monthly means that varied from 0.7 ± 0.3 to 73.3 ± 9.3 flies per animal (Figure 2(c)). The horn fly population was lower in the first six months of 2008 and considerably higher in the first six months of 2009. The months of greatest population were from September to January 2009 and from April to May 2009, with population peaks in April and October. The mean burdens of* H. irritans* did not exhibit a significant correlation (*P* > 0.05) with any climatic parameter (Table 1).

Despite the low mean levels of parasites in the herd, emergency treatments were applied three times on animals from group A: July 2008 (1 animal), August 2008 (2 animals), and September 2008 (1 animal) and twice on group B: July 2008 (1 animal) and December 2008 (1 animal) because of high tick infestations; further treatments were applied twice on animals from group A: August 2009 (1 animal) and September 2009 (1 animal) and three times on group B: February 2009 (1 animal), August 2009 (2 animals), and September 2009 (2 animals) due to botfly larvae. There was no need of emergency treatments for* H. irritans*. Such treatments reflect the irregular distribution of parasites in the animals of the herd.

**Figure 3:**
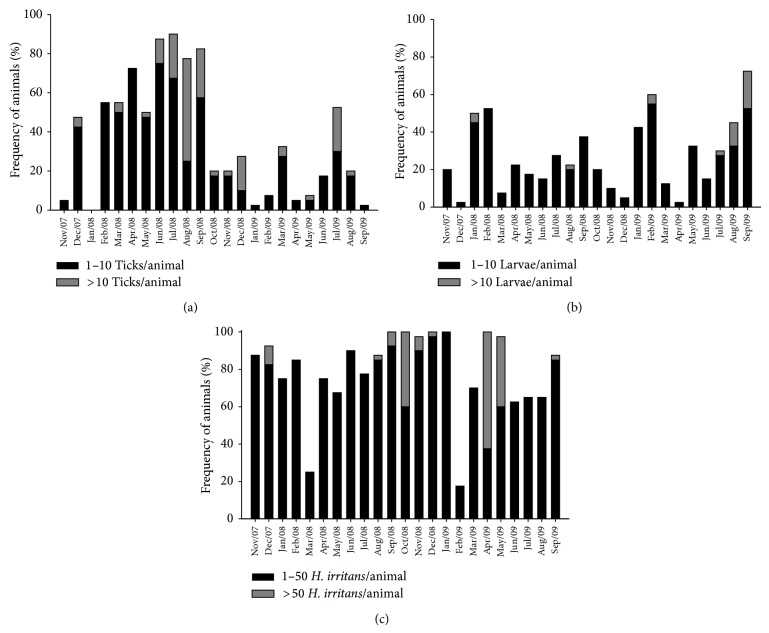
Frequency distribution of* Rhipicephalus microplus* (a) and larvae of* Dermatobia hominis* (b) and* Haematobia irritans* (c) on heifers from Holstein × Gir crossbreeds from November 2007 to September 2009 in the northeast of Minas Gerais State, Brazil.

Analysis of the distribution of parasites on experimental animals during all months of the year revealed that the percentage of animals with more than 10 ticks ranged from 0 to 25%, with the sole exception of August 2008, when 52.5% of the animals harbored more than 10 ticks (Figure 3(a)). During 15 months of the experiment, more than 50% of the animals were free of ticks.

The percentage of animals of the herd with* D. hominis* larvae ranged from 2.5 to 55% (Figure 3(b)). In 15 months, fewer than 30% of the animals were responsible for the entire parasitic load of* D. hominis*, and during 20 of the 23 months of the trial, more than 50% of the herd was free of larvae. Only 12 animals (30%) accounted for 76.5% of the larvae observed during the study.

The frequency data of infested animals showed that the horn fly was the only ectoparasite able to infest all of the animals in the herd, with all animals harboring this fly in the months of September, October, and December of 2008 and January and April of 2009 (Figure 3(c)). The flies were present on more than 60% of animals in all months except March 2008 and February 2009. In the months during which higher populations were observed, horn fly loads of more than 50 flies per animal were observed on up to 62.5% of the animals in the herd.

The overall median of ticks per animal was zero from both groups A and B and there was no significant difference between groups A and B in any month of the trial (*P* > 0.05) (Table 2). The median number of* D. hominis* larvae for group A (1/4* B. taurus*) was 0.0 (0-0), whereas for group B was 0.0 (0–3) (Table 2). There was a significant difference (*P* < 0.05) between the densities of the parasitic groups for seven months of the study, with the density always being higher on 1/2 Holstein animals.

Group A had a significantly lower* H. irritans* density (*P* < 0.05) than group B, with medians of 3.0 (1–11) and 6.0 (1–25) flies per animal, respectively (Table 2). In every month during which the mean horn fly load exceeded three flies/animal, group B was more infested than group A, and this difference was statistically significant (*P* < 0.05) in four of the months evaluated.

## 4. Discussion

The ectoparasites* R. microplus, D. hominis, *and* H. irritans* are among the primary species that cause economic damage to cattle in Brazil. The climatic conditions in most areas of the country, including MG, are considered favorable for the development and maintenance of parasites throughout the entire year.

In the studied area, the mean numbers of parasites in the herd were considered low, nevertheless some animals exceeded the limit of parasites stipulated (≥40 ticks, ≥20 botfly nodules or ≥200 horn flies) and needed an emergency treatment. To avoid interference in the epidemiology of the parasites during the experimental period and to enable the maintenance of treated animals in the experiments, treatments were performed with trichlorphon (a short-acting ectoparasiticide) after the parasite count from each month [7, 8]. When used in the formulation above (0.2% in water applied topically with a brush), parasites on the animals were killed, but the residual effect was consistently reduced and did not prevent the animals from harboring parasites in subsequent counts. The emergency treatments may have subtly reduced the number of parasites in the environment, on an insufficient level to affect the population dynamics of ectoparasites in the present study. Such conclusion is achieved once the treatments were performed only in seven out of 23 months and always in a small number of animals (2.5 to 7.5% of the flock) while, during these months, the frequency of parasitized animals in the herd ranged from 27.5 to 90% for ticks (with 17.5 to 25% with >10 ticks) and 45 to 72.5% for botfly (with 5 to 20% with >10 larvae).

The levels of parasitism by* R. microplus* were low, with the highest density of parasitism in midwinter (July-August), a period that is characterized by lower rainfall and lower temperatures. Temperature was the only climatic factor that correlated significantly (*P* < 0.05) with the population levels, indicating that the warm period, when the mean maximum temperature always exceeds 28°C, was unfavorable to the development and/or survival of tick larvae in the pasture. These results are in contrast to the findings from other regions of the state with lower maximum and minimum temperatures (such as Florestal, MG), where the factor that most influenced the dynamics of ticks was the rainfall [9].

The low tick burdens found in the present study (mean of 3.0 ticks per animal) were similar to those found by Oliveira and Alencar [10] in Holstein × Guzera crossbreeds in São Carlos (São Paulo State) when the mean parasite loads were 1.76 and 2.79 for 1/4 and 1/2 Holstein cattle, respectively.

In all experimental months, fewer than 30% of the animals exhibited a parasite load of more than 10 ticks, with the exception of August 2008, which was the peak of parasitism. These results corroborate the findings of other studies in which less than 25% of the herd harbored more than 45% of the ticks [9, 11]. There was no significant difference between the tick loads of groups A and B, most likely due to the low parasite loads or to the small difference in the genetics of the animals, which was only 1/4; however, other similar studies have demonstrated greater susceptibility of* B. taurus* breeds to ticks [9, 10].

The seasonal variations in the populations of adult* H. irritans* and larval* D. hominis* on cattle showed that both populations had two peaks during the year. One similarity between these ectoparasites was that both had greater influence from rainfall on the seasonal dynamics. These two species also had higher population levels in the last twelve months of the trial, confirming that both parasites are supported by similar climatic conditions.

The mean number of* D. hominis* larvae per animal had a significant negative correlation (*P* < 0.05) with the amount of rainfall, indicating that the high levels of rain in some months hampered parasite infestation. These results are relatively similar to those of studies performed in the states of Mato Grosso do Sul (MS) and Rio de Janeiro (RJ), where the annual population peaks were observed in March, May, and August-September and where the rainfall was the climate parameter that most influenced the population levels [12–14].

The mean levels of* D. hominis* larvae on the animals (1.5 larvae per animal) were lower than those observed in studies conducted in the Brazilian Cerrado, where means of 18.4 to 22.6 larvae/animal were recorded on* B. taurus*,* B. indicus,* and crossbred animals [10, 12]. In the present study, 30% of the animals of the herd harbored 76.5% of the larvae.* D. hominis* was the parasite that exhibited the greatest difference between groups A and B; the mean number of* D. hominis* larvae on 1/2 Holstein ×  1/2 Gir cattle was 5.5 times higher than that on 1/4 Holstein ×  3/4 Gir animals, and this difference was significant (*P* < 0.05) for seven experimental months. Previous work has provided evidence of individual variation in ectoparasite susceptibility among animals of the same genetic group and has showed that, in general, the higher proportion of* B. taurus* genetic make-up lead to a higher susceptibility to* D. hominis* larvae [10, 12]. The differences between the 1/4 and 1/2 Holstein × Gir animals used in the present study were higher than those observed by Oliveira and Alencar [10] for 1/4 and 1/2 Holstein × Guzera animals, which harbored 4.18 and 4.34 larvae per animal, respectively.

*H. irritans* was the most prevalent ectoparasite throughout the study. It was the only species present on animals in all months of the year and was also the parasite that parasitized all animals of the herd in the months of the population peaks. In addition to the population peaks observed in the spring, the period from November to March (late spring and entire summer) was characterized by intermediate levels of infestation followed by a second population peak in April-May (midautumn). The population dynamics of the horn fly vary during the year due to climatic factors. The periods with the highest population usually occur 1–3 weeks after rains [15], and temperatures approximately 25°C and humidity above 65% are optimal conditions for development, whereas low temperatures can induce diapause [16], and dry periods may increase the mortality of larvae in the stool [17]. In the studied area, the temperature fluctuations were small, and rainfall was most likely the climatic parameter that most influenced the population dynamics, although the correlation with rainfall was not statistically significant (*P* > 0.05). Similar results were observed in other regions of Brazil. Lima et al. [18], in the state of São Paulo (SP), and Barros [19], in the Mato Grosso Pantanal region, also observed population peaks in the spring and autumn (the beginning and end of the rainy season). Research carried out in Southern Brazil and Argentina showed that the temperature, and not the rainfall, is the climatic factor that most influences the population level in temperate regions [20, 21].

Torrential rains are frequent in the studied area and may explain the differences in population levels of* H. irritans* between the two years of the study. Previous works have showed that approximately 100 mm of rain in one week could cause a reduction in the population by dispersing the stool and preventing the development of larvae [15, 22]. The frequency of animals in the herd with horn flies was similar to the frequency of animals with other parasites. Fewer than 40% of the animals in the flock harbored 50 or more flies in 22 of the 23 months. Previous research showed that the mean number of flies on susceptible animals can be more than twice the number on resistant animals [23]. The mean number of flies/animal did not exceed 80; the value is similar to the values obtained in other studies by Bianchin and Alves [24] and Bianchin et al. [25] but is lower than the values observed in other studies conducted in Brazil [18] and Argentina [26].

A higher parasite load was observed in group B, which had the highest degree of* B. taurus* genetic make-up (1/2 Holstein) and whose parasite load was statistically higher (*P* < 0.05) in four months of the study. These results corroborated the findings of several studies performed in different countries of South America, where horn flies reach higher densities in* B. taurus* breeds such as Hereford, Angus, and Holstein compared to zebu breeds such as Nelore, Guzera, and Brahman [6, 23, 26, 27].

A program that is aimed at controlling ectoparasites using insecticides and/or acaricides must address bioecological aspects and population dynamics of parasites in order to limit actions to a few per year, thereby reducing the number of treatments, costs, manpower, and the use of ectoparasiticide compounds and their residues in animal products. In this study, the levels of parasitism for* R. microplus* were low and if considered alone, it would be unlikely to inflict great damage on the production of the herd. Meanwhile, the levels of* D. hominis* and* H. irritans* were considerably higher and could be responsible for damage and concern. Previous studies show that parasitic loads of 20–40 larvae of* D. hominis* per animal can cause 9–14% reduction in weight gain, and parasitic loads of more than 50 larvae cause 18–25% reduction in the milk production [28]. For* H. irritans*, less than 50 adults per animal can reduce weight gain by 8.6–16%, depending on the age of the animal [29]. According to our results, the most appropriate times to apply ectoparasiticides in the region of study are August-September (late dry season) and April-May (end of the rainy season), which are the months before the parasite population peaks, as previously suggested by other authors [30–33]. Application at the end of the rainy season aims to reduce the population as a whole because these compounds combat parasites on the animals, whereas those in the environment face poor living conditions in the pastures [31]. A supplementary application could be performed in the middle of the rainy season (in December or January) to reduce the parasite populations at the beginning of the summer season.

Another possible strategy to effectively control the parasite population consists of directing the treatment to a few animals of the herd. Our data showed that a few animals in the herd harbor most of the parasites, and when the ectoparasite population increases, most of the new specimens are housed in animals with a higher proportion of* B. taurus* genetic make-up, despite the relatively small difference in genetic make-ups between the studied groups (only 1/4 Holstein). Our data suggest that these susceptible animals could be the focus of treatment programs and may be the only ones to receive ectoparasiticide treatment. The percentage of animals to be treated varies according to the frequency of distribution of parasites in the herd, which depends mainly on the cattle breed, season, and ectoparasite species. Different percentages were used in previous work, ranging from 4.9 to 54.3% for* R. microplus* [34, 35] and 33.3% for* H. irritans* [36]. All of them resulted in reduction in the level of parasites on the animals and economic benefits.

## Figures and Tables

**Table 1:** Spearman's coefficients for the correlations between the climatic parameters and the ectoparasite counts.

Variable	*R. microplus *	*D. hominis *	*H. irritans *
Pluviometry	−0.1929	−0.3924∗	0.0158
Maximum temperature	−0.3306	−0.0448	−0.1847
Minimum temperature	−0.3734∗	−0.1933	−0.2201

^*^Significant correlation (*P* < 0.05) between variables.

**Table 2:** Ectoparasite counts on heifers from Holstein × Gir crossbreeds from November 2007 to September 2009 in the northeast of Minas Gerais State, Brazil.

Month	*R. microplus *		*D. hominis *		*H. irritans *	
	Group A	Group B	Group A	Group B	Group A	Group B
Nov./07	0.0 (0.0) 0-0	0.0 (0.3) 0-0	0.0 (0.4) 0–0.3	0.0 (0.0) 0-0	0.0 (4.2) 1–6	6.5 (8.5) 3–11.8

Dec./07	0.0 (2.1) 0–2.5	3.0 (4.3) 0–6.5	0.0 (0.2) 0-0	0.0 (0.0) 0-0	10.5 (15.7) 5.5–19.5	13.5 (29.5) 3.8–31.8

Jan./08	0.0 (0.0) 0-0	0.0 (0.0) 0-0	0.0 (1.0) 0-1	∗2.0 (5.5) 1.5–7	0.0 (2.1) 0–3	2.5 (3.1) 1–4

Feb./08	1.0 (1.7) 0–3	0.0 (0.4) 0-1	0.0 (0.7) 0-1	1.0 (2.9) 0.8–4.3	1.0 (1.7) 1–2.3	2.0 (2.0) 1-2

Mar./08	2.0 (3.4) 0–6	0.0 (0.8) 0–2	0.0 (0.1) 0-0	0.0 (0.1) 0-0	0.0 (0.9) 0–1.3	0.0 (0.9) 0-0

April/08	2.0 (2.6) 0.8–4	1.0 (1.5) 0–2.5	0.0 (0.2) 0-0	0.0 (0.9) 0–1.3	1.0 (1.5) 1-2	2.0 (2.8) 0–2.3

May/08	1.0 (1.8) 0–2	1.0 (2.3) 0–3	0.0 (0.2) 0-0	0.0 (1.4) 0–1.5	1.0 (1.7) 0–2	1.0 (5.0) 0–3

June/08	3.0 (4.7) 1.8–5.5	3.5 (4.4) 1–5.8	0.0 (0.2) 0-0	0.0 (0.5) 0–0.3	2.5 (2.9) 1–4	4.5 (8.1) 2–9

July/08	5.0 (7.6) 2–8.5	4.0 (6.5) 1.5–8.5	0.0 (0.3) 0-0	∗2.5 (3.0) 0–4.3	2.0 (3.6) 0.8–5.3	∗8.0 (10.5) 3.8–15

Aug./08	15.0 (18.1) 4–21	∗0.0 (8.5) 0–7	0.0 (0.4) 0-0	∗3.5 (5.1) 0–8.5	3.5 (4.5) 1.8–6.3	6.0 (17.6) 2–8

Sept./08	8.0 (9.4) 3–12	1.5 (5.3) 0–8	0.0 (0.5) 0-1	1.5 (2.9) 0–5.5	23.5 (25.2) 14.8–34.5	35.5 (43.1) 26.8–53.8

Oct./08	0.0 (0.8) 0-0	0.0 (2.0) 0-0	0.0 (0.5) 0-0	0.0 (0.8) 0-0	40.0 (45.3) 23.5–61.8	67.0 (67.9) 47–92.5

Nov./08	0.0 (2.1) 0-0	0.0 (0.3) 0-0	0.0 (0.3) 0-0	0.0 (0.0) 0-0	9.0 (13.0) 5–23	∗32.5 (35.6) 10.5–53.3

Dec./08	0.0 (3.4) 0-0	1.0 (9.5) 0–12	0.0 (0.1) 0-0	0.0 (0.1) 0-0	9.0 (12.3) 5–18.5	17.5 (21.9) 6.8–26.3

Jan./09	0.0 (0.2) 0-0	0.0 (0.0) 0-0	0.0 (1.0) 0-1	2.0 (2.5) 0–3.3	9.0 (12.5) 5–14.3	14.0 (17.1) 7–26.5

Feb./09	0.0 (0.3) 0-0	0.0 (0.8) 0-0	0.5 (1.8) 0–3	∗5.0 (8.0) 3.8–7.3	0.0 (0.7) 0-0	0.0 (0.6) 0–0.5

Mar./09	0.0 (2.1) 0-0	0.0 (4.0) 0-1	0.0 (0.2) 0-0	0.0 (0.0) 0-0	1.0 (1.5) 0–2	∗6.0 (5.8) 5.5–6.3

April/09	0.0 (0.4) 0-0	0.0 (0.0) 0-0	0.0 (0.0) 0-0	0.0 (0.0) 0-0	59.5 (68.2) 42.3–75.3	90.0 (93.8) 41–143.3

May/09	0.0 (0.8) 0-0	0.0 (0.0) 0-0	0.0 (0.5) 0-1	0.5 (0.8) 0–1.3	32.0 (45.1) 18.3–57	∗82.5 (79.5) 42.3–103.8

June/09	0.0 (0.8) 0-0	0.0 (0.0) 0-0	0.0 (0.1) 0-0	0.0 (0.4) 0-1	0.0 (2.7) 0–3.3	6.0 (6.4) 0.8–10

July/09	5.0 (7.7) 0–10.5	0.0 (3.1) 0–5	0.0 (0.9) 0-0	∗5.0 (8.4) 0.5–7.5	1.0 (2.2) 0–3	0.0 (6.4) 0–2.5

Aug./09	0.0 (1.0) 0-0	0.0 (0.9) 0-0	0.0 (2.5) 0–1.5	∗20.0 (32.7) 6.5–55.5	2.0 (2.6) 0.5–3	0.0 (0.4) 0-1

Sept./09	0.0 (0.1) 0-0	0.0 (0.0) 0-0	3.0 (7.4) 1–7.5	∗12.0 (32.1) 7–37	7.0 (8.5) 3–12	5.0 (21.3) 3–24

Total	**0.0 (3.1)** **0–3**	**0.0 (2.4)** **0–2**	**0.0 (0.8)** **0-0**	**0.0 (4.4)** **0–3**	**3.0 (12.1)** **0.9–24.2**	**6.0 (21.4)** **2.6–35.6**

Group A: 1/4 Holstein × 3/4 Gir; Group B: 1/2 Holstein × 1/2 Gir.

Data are presented as median (mean) quartile 1–quartile 3.

∗Significant difference between groups A and B (*P* < 0.05; Mann-Whitney *U* test).
